# A Point Prevalence Survey of antibiotics use patterns at Tanga Regional Referral Hospital in Tanzania

**DOI:** 10.1017/ash.2025.10199

**Published:** 2025-10-20

**Authors:** Aneth Wilbroad Mutatina, Ernest Moses Lomnyak, Thomas Bizimana, Emiliana Nyafungo Francis, Daudi Msasi, Zalha Elsalmy Nuhu, Emmanuel Magembe, Raphael Zozimus Sangeda

**Affiliations:** 1 Pharmaceutical Services Unit, Ministry of Health, Dodoma, Tanzania; 2 Department of Maritime Transport, Dar es Salaam Maritime Institute (DMI), Dar es Salaam, Tanzania; 3 East African Center of Excellence for Vaccines, Immunisation and Health Supply Chain Management, University of Rwanda, Kigali, Rwanda; 4 Tanga Regional Referral Hospital, Tanga, Tanzania; 5 National Public Health Laboratory, Dar es Salaam, Tanzania; 6 Department of Pharmaceutical Microbiology, Muhimbili University of Health and Allied Sciences, Dar es Salaam, Tanzania

## Abstract

**Objective:**

Antimicrobial resistance (AMR) is a major global health threat, driven in part by inappropriate antibiotic use. This study assessed the prevalence, patterns, and determinants of antibiotic prescription among inpatients at Tanga Regional Referral Hospital (RRH), Tanzania, using the World Health Organization (WHO) Point Prevalence Survey (PPS) methodology.

**Design:**

A cross-sectional PPS was conducted using the WHO tool. Descriptive statistics summarized prescribing patterns, and logistic regression identified factors associated with antibiotic use.

**Setting:**

Tanga RRH in Tanzania among hospitalized patients in April 2023.

**Results:**

Among 205 inpatients (60.5% female), 77.6% (*n* = 159) received ≥1 antibiotic, with a mean of 2.1 agents per patient. Metronidazole (27.7%) and ceftriaxone (17.6%) were the most frequently prescribed antibiotics. Only 2.4% of prescriptions were supported by antimicrobial susceptibility testing. In multivariate analysis, the absence of a peripheral vascular catheter was independently associated with reduced odds of antibiotic use (aOR 0.16, 95% CI: 0.06–0.43). Although 89% of prescriptions followed the Tanzanian Standard Treatment Guidelines, prescribing patterns diverged from WHO stewardship targets: only 53.8% of antibiotics were from the Access group (below the ≥60% recommended threshold). In comparison, 44.4% belonged to the broader-spectrum Watch group.

**Conclusion:**

Antibiotic use in Tanga RRH was high, with limited microbiological guidance and heavy reliance on broad-spectrum Watch antibiotics, highlighting stewardship gaps. Strengthening antimicrobial stewardship and expanding diagnostic capacity are urgently needed to optimize antibiotic use and curb AMR in resource-limited settings.

## Introduction

Antimicrobial resistance (AMR) has emerged as a pressing global health threat, driven largely by the inappropriate use of antibiotics in healthcare settings.^1,2^ The misuse and overuse of antibiotics accelerates the development of resistant pathogens, undermining the effectiveness of existing treatments, and placing an increasing burden on healthcare systems.^3^

In response, the World Health Organization (WHO) introduced a standardized methodology known as the Point Prevalence Survey (PPS) to collect and harmonize data on antimicrobial use across healthcare institutions.^4^ This tool enables hospitals to assess prescribing practices, identify gaps in antimicrobial stewardship (AMS), and guide interventions. PPS data from high-income countries (HICs) show relatively low antibiotic use compared to the higher usage rates reported in low- and middle-income countries (LMICs), particularly in West and Northern Africa.^5–9^

Tanzania officially launched its national action plan (NAP) for the AMS program in 2018.^10^ During NAP implementation, some WHO PPS in Tanzania reported average antibiotic use rates ranging from 44% to 62.3%.^11^ Although follow-up assessments have demonstrated incremental progress in AMS, many facilities still lack systematic surveillance of antimicrobial use, particularly in relation to the PPS methodology.^12^

To address the gap in the implementation of the NAP-AMS program, the present study applied the WHO PPS methodology to assess the prevalence, type, and indications of antibiotic use, identify factors associated with prescribing, and evaluate adherence to standard treatment guidelines (STGs) among inpatients at Tanga Regional Referral Hospital (RRH). These findings aim to inform local AMS efforts and contribute to a broader national strategy for AMR containment in Tanzania and globally.

## Materials and methods

### Study design and setting

A quantitative descriptive cross-sectional study was conducted using the WHO PPS methodology. The study was conducted at Tanga RRH, the highest-ranking referral hospital in the Tanga region and one of the NAP-AMS implementing hospitals in Tanzania. The Tanga region is one of the 26 administrative regions of Tanzania’s mainland, located in northeastern Tanzania. The region has a population of approximately 2,615,597. Tanga RRH provides services to patients referred from 18 district hospitals, 74 health centers, and 693 dispensaries.

### Study population

The target population was patients admitted to Tanga RRH. The hospital has a total bed capacity of 457 beds, with an average annual admission rate of 13,000. The study was conducted between 18^th^ April and 20^th^ April 2023.

According to the WHO PPS methodology, all patients admitted to each ward before 8:00 am on the survey day were included in the study, regardless of sex and age. All antibiotics administered via inhalation and oral, rectal, and parenteral routes were included. The study did not include patients admitted to the emergency department and renal dialysis, discharged patients, patients waiting for bill clearance or transport, patients on topical and ophthalmic antibiotics and patients with incomplete medical records.^4^

### Sampling procedures and sample size

Tanga RRH was conveniently selected among the RRHs implementing AMR programs that had not established antimicrobial utilization using the WHO PPS tool.

The WHO PPS methodology required the inclusion of all patients in the hospital who met the criteria if their bed capacity was less than 500.^4,11^

### Data collection instruments and collection procedures

To assess the quality of antibiotic use, this study followed the WHO PPS methodology (WHO, 2018), which includes indicators related to the appropriateness of antibiotic prescription. These indicators include documentation of the indication for antibiotic therapy, adherence to national STGs,^13^ availability of stop or review dates, and the use of microbiological testing to guide therapy, such as antimicrobial susceptibility testing (AST). This study evaluated guideline adherence by comparing each prescribed antibiotic with the Tanzanian STG.^13^ For each prescription, data collectors assessed whether the indication and treatment matched the national guideline recommendations for diagnosis, dose, route, and frequency. Additionally, prescriptions were classified under the WHO AWaRe (Access, Watch, Reserve) categorization to provide a proxy measure of stewardship quality. Documentation of the indication for treatment, the presence or absence of culture samples and AST, and whether a stop or review date was recorded were also extracted. These serve as core indicators of quality in prescribing. Specifically, empirical prescribing was inferred where no AST was performed and where documentation indicated empiric therapy. The proportion of antibiotics prescribed empirically versus those guided by culture results was also used as an indirect indicator of quality.

The personnel involved in data collection were pharmacists and clinical officers trained in the WHO PPS methodology. The training included the interpretation of PPS forms, understanding clinical documentation, and applying national STGs. Data were validated by a senior clinical pharmacist before entry into the WHO PPS Excel tool and later reviewed again during data analysis.

Data were collected using the WHO PPS Excel tool. Data were extracted from the patients’ medical records. The data collection exercise lasted for two consecutive weeks, whereby each ward was completed in one day to reduce the impact of patients’ ward transfer and discharge.^4^

Other data collected included hospital-level information, including bed capacity, annual admission, and patient characteristics; ward-level data included ward type and number of patients who met criteria; patient-level data included patient age, sex, catheter presence, surgery, antibiotic use, and hospitalization; indication-level data included indication for antibiotic use and laboratory tests; antibiotic-level data included antibiotic type, strength, administration route, frequency, and oral switch status.

### Data entry, analysis, and presentation

Following data validation, the data set from the WHO PPS Excel tool was exported and analyzed using IBM SPSS Statistics version 26. Descriptive statistics, including frequencies and proportions, were used to summarize antibiotic prescribing patterns by patient demographics, ward characteristics, and clinical indicators.

Prescribing quality was assessed based on documented indications, compliance with the Tanzanian STG,^13^ AST, and classification under the WHO AWaRe framework. Antibiotic use was also categorized as empirical or targeted, where applicable.

Univariate logistic regression was conducted to explore the associations between antibiotic use and clinical or demographic variables. Variables with a significance level of *P*-value ≤ .02 were entered into a multivariate logistic regression model to identify independent predictors. Adjusted odds ratios (aORs) with 95% confidence intervals (CIs) were calculated and reported.

### Ethical considerations

Ethical clearance was pursued and awarded with reference No. HD.209/247/07A after obtaining the recommendation letter for ethical approval and data collection from the University of Rwanda. The Permanent Secretary, Ministry of Health, and the Medical Officer in charge of Tanga RRH gave authorization to conduct the study in the hospital. This study collected patient information from medical records without direct interaction with patients. Therefore, informed consent was not required in this study. Confidentiality was assured by assigning each patient a unique, anonymous code to hide their identity. Study laptops were protected to ensure that only authorized personnel could access the data.

## Results

### Demographic information

Of 219 patients hospitalized during the study period, 205 were included in the analysis. The hospital had a total bed capacity of 457. Of the participants, 60.5% (*n* = 124) were female, and the predominant age group was 20 – 64 years, representing 59.0% (*n* = 121) of the patients. The neonatal medical ward had the highest patient contribution at 21.0% (*n* = 43), followed by the adult medical (28.8%) and adult surgical wards (31.2%). Most of the patients (80.5%, *n* = 165) had a peripheral vascular catheter and 27.3% (*n* = 56) had a urinary catheter in place (Table 1).

**Table 1.** Demographic and clinical characteristics of hospitalized patients at Tanga Regional Referral Hospital (*n* = 205), stratified by antibiotic use status

Demographic and clinical variables		Overall frequency *N* (%)	Not on antibiotics (*n* = 46) *N* (%)	On antibiotics (*n* = 159) *N* (%)	^*^ *p*-value
Age (years)					.482
	Less than 2	50 (24.4)	13 (26.0)	37 (74.0)	
	2 – 5	4 (2.0)	2 (50.0)	2 (50.0)	
	6 – 12	4 (2.0)	1 (25.0)	3 (75.0)	
	13 – 19	13 (6.3)	4 (30.8)	9 (69.2)	
	20 – 64	121 (59.0)	25 (20.7)	96 (79.3)	
	65 and above	13 (6.3)	1 (7.7)	12 (92.3)	
Gender					.687
	Female	124 (60.5)	29 (23.4)	95 (76.6)	
	Male	81 (39.5)	17 (21.0)	64 (79.0)	
Wards					**<.001**
	Neonatal medical	43 (21.0)	13 (30.2)	30 (69.8)	
	Adult medical	59 (28.8)	23 (39.0)	36 (61.0)	
	Adult surgical	64 (31.2)	7 (10.9)	57 (89.1)	
	Pediatric medical	8 (3.9)	0	8 (100.0)	
	Adult intensive care unit	5 (2.4)	1 (20.0)	4 (80.0)	
	Mixed ward	26 (12.7)	2 (7.7)	24 (92.3)	
Surgery since admission					**<.001**
	Yes	62 (30.2)	1 (1.6)	61 (98.4)	
	No	142 (69.3)	45 (31.7)	97 (68.3)	
	Unknown	1 (.5)	0	1 (100.0)	
Central vascular catheter					.577
	Yes	4 (2.0)	0	4 (100.0)	
	No	201 (98.0)	46 (22.9)	155 (77.1)	
Peripheral vascular catheter					**<.001**
	Yes	165 (80.5)	23 (13.9)	142 (86.1)	
	No	40 (19.5)	23 (57.5)	17 (42.5)	
Urinary catheter					**<.001**
	Yes	56 (27.3)	1 (1.8)	55 (98.2)	
	No	146 (71.2)	45 (30.8)	101 (69.2)	
	Unknown (UC)	3 (1.5)	0	3 (100.0)	
Intubation					.601
	Yes	2 (1.0)	0	2 (100.0)	
	No (Intubation)	203 (99.0)	46 (22.7)	157 (77.3)	
Hospitalization within 90 Days					.653
	Yes	25 (12.2)	4 (16.0)	21 (84.0)	
	No	164 (80.0)	38 (23.2)	126 (76.8)	
	Unknown	3 (1.5)	1 (33.3)	2 (66.7)	
	Missing	13 (6.3)	^**^	^**^	
Malaria status			^**^	^**^	
	Yes	8 (3.9)	^**^	^**^	
	No	21 (10.2)	^**^	^**^	
	Unknown	165 (80.5)	^**^	^**^	
	Missing	11 (5.4)	^**^	^**^	
Tuberculosis status			^**^	^**^	
	Yes	2 (1.0)	^**^	^**^	
	No	12 (5.9)	^**^	^**^	
	Unknown	167 (81.5)	^**^	^**^	
	Missing	24 (11.7)	^**^	^**^	
HIV status			^**^	^**^	
	Yes	11 (5.4)	^**^	^**^	
	No	29 (14.1)	^**^	^**^	
	Unknown	139 (67.8)	^**^	^**^	
	Missing	26 (12.7)	^**^	^**^	

* *P*-values represent comparisons between patients on antibiotics and those not on antibiotics within each subgroup. χ^2^ tests were used unless the expected cell counts were <5, in which case Fisher’s exact test was used. Statistically significant associations (*P* < .05) are shown in bold for ward type, surgery since admission, peripheral vascular catheter, and urinary catheter.

** The proportion of patients on antibiotics was not categorized by malaria, tuberculosis (TB), or HIV status due to the high proportion of missing or unknown data.

### Prevalence of use and association with patient characteristics

Of the 205 hospitalized patients, 159 (77.6%) were receiving antibiotic treatment, with a mean of 2.1 antibiotics per patient. Among those on antibiotics, 104 (65.4%) were prescribed two agents, 25 (15.7%) received one, 26 (16.4%) received three, and only a few received four (2 patients, 1.3%) or five (2 patients, 1.3%).

Antibiotic use varied across clinical characteristics. Higher proportions were observed among patients admitted to adult surgical, pediatric medical, and mixed wards (*P* < .001). Antibiotic use was also more common among patients who had undergone surgery since admission (*P* < .001) and among those with peripheral vascular catheters (*P* < .001) or urinary catheters (*P* < .001). No significant differences in antibiotic use were noted by age group, gender, intubation status, or hospitalization within the previous 90 days (Table 1).

### Distribution of the route and dose frequency

Among the 329 antibiotic encounters recorded in 159 patients, the majority were administered parenterally (86.0%, *n* = 291), whereas 14.0% (*n* = 49) were administered orally. A subset (11.6%, *n* = 38) involved switching from parenteral to oral administration. Regarding dosing frequency, most antibiotics were prescribed three times per day (44.7%), followed by twice daily (38.3%), once daily (16.4%), and rarely four times daily (0.6%).

### Antibiotic use frequency

A total of 329 antibiotic encounters were recorded, with the most common being metronidazole (27.7%, *n* = 88) and ceftriaxone (17.6%, *n* = 58) (Table 2). Together with gentamicin, ampicillin + cloxacillin, ampicillin, and cefotaxime, these six antibiotics accounted for over 90% of all prescriptions, consistent with the drug utilization 90% (DU90) metric. The main prescribed pharmacological groups were imidazoles (28%), penicillins (26%), and cephalosporins (25%).

**Table 2.** Percentage and cumulative distribution of individual antibiotic use (*n* = 329 encounters) at Tanga Regional Referral Hospital, Tanzania

Antibiotic	*N* (%)	Cumulative %
Metronidazole	88 (26.7)	26.7
Ceftriaxone	58 (17.6)	44.4
Gentamicin	50 (15.2)	59.6
Ampicillin + cloxacillin	43 (13.1)	72.6
Ampicillin	33 (10.)	82.7
Cefotaxime	27 (8.2)	90.9
Amoxicillin + clavulanic acid	8 (2.4)	93.3
Clarithromycin	6 (1.8)	95.1
Meropenem	4 (1.2)	96.4
Erythromycin	3 (.9)	97.3
Sulfamethoxazole + trimethoprim	2 (.6)	97.9
Clindamycin	2 (.6)	98.5
Ciprofloxacin	2 (.6)	99.1
Ceftizoxime	1 (.3)	99.4
Tinidazole	1 (.3)	99.7
Flucloxacillin + ampicillin	1 (.3)	100

Among the 329 antibiotic encounters, 89.1% (*n* = 293) complied with the Tanzanian STGs. When categorized according to the WHO AWaRe classification, 53.8% of prescriptions were from the Access group, 44.4% from the Watch group, and 1.8% from the Reserve group (Table 3).

**Table 3.** Distribution of prescribed antibiotics by WHO AWaRe classification and pharmacological class (*n* = 329 encounters) at Tanga regional referral hospital, Tanzania

Aware Class		*N*(%)
	Access	177 (53.8)
	Watch	146 (44.4)
	Reserve	6 (1.8)
**Antibiotic group**		
	Imidazoles	89 (27.1)
	Cephalosporins	86 (26.1)
	Penicillins	84 (25.5)
	Aminoglycosides	50 (15.2)
	Macrolides	11 (3.3)
	Carbapenems	4 (1.2)
	Fluoroquinolones	3 (.9)
	Sulfonamides	2 (.6)

### Indications for antibiotic use and antibiotic susceptibility testing

Of 170 recorded antibiotic indications, nearly half (47.6%) were for community-acquired infections, followed by 30.6% categorized as “Other.” Surgical prophylaxis and hospital-associated infections each accounted for 9.4% of the indications, while medical prophylaxis was the least common (2.9%). Despite these indications, only four (2.4%) were linked to culture samples taken for AST, and only one isolate, *Escherichia coli*, had a documented result.

### Factors associated with antibiotic use

Of the 205 patients, univariate logistic regression identified several factors significantly associated with antibiotic use. Compared with patients in adult medical wards, those in adult surgical wards had markedly higher odds of receiving antibiotics (OR: 5.202, 95% CI: 2.025 – 13.363, *P* = .001). Admission to mixed wards (OR: 7.667, 95% CI: 1.653 – 35.564, *P* = .009) was also associated with increased antibiotic use. In contrast, patients who had not undergone surgery since admission (OR: 0.035, 95% CI: 0.005 – 0.263, *P* = .001) and those without peripheral vascular catheters (OR: 0.120, 95% CI: 0.056 – 0.258, *P* < .001) or urinary catheters (OR: 0.041, 95% CI: 0.005 – 0.304, *P* = .002) were significantly less likely to be prescribed antibiotics.

In the multivariate model, only the absence of a peripheral vascular catheter remained significantly associated with reduced odds of antibiotic use (aOR: 0.163, 95% confidence interval [CI], 0.063 – 0.426; *P* < .001). Other variables—including ward type, gender, surgery since admission, and urinary catheter use—were retained for clinical relevance but were not statistically significant after adjustment (Table 4).

**Table 4.** Factors associated with antibiotic use among hospitalized patients at Tanga regional referral hospital, Tanzania (univariate and multivariate logistic regression, *n* = 205)

Variable	Crude OR (95% CI)	*p*-value	Adjusted OR (95% CI)	*p*-value
**Ward**				
** Adult Medical Ward**	Reference	–	Reference	–
** Adult Surgical Ward**	5.202 (2.025 – 13.363)	.001	.989 (.241 – 4.055)	.987
** Neonatal Ward**	1.474 (.640 – 3.398)	.362	4.275 (.780 – 23.424)	.094
** Mixed Ward**	7.667 (1.653 – 35.564)	.009	–	.997^*^
** ICU**	2.556 (.269 – 24.317)	.414	–	.999^*^
** Pediatric Medical Ward**	–	–	–	.999^*^
**Gender**				
** Male**	Reference	–	Reference	–
** Female**	.870 (.442 – 1.713)	.687	.755 (.292 – 1.952)	.562
**Age group**				
** <2 years**	Reference	–	–	–
** 2 – 5 years**	.351 (.045 – 2.755)	.319	–	–
** 6 – 12 years**	1.054 (.101 – 11.049)	.965	–	–
** 13 – 19 years**	.791 (.208 – 3.009)	.730	–	–
** 20 – 64 years**	1.349 (.625 – 2.914)	.446	–	–
** ≥65 years**	4.216 (.498 – 35.679)	.187	–	–
**Surgery since admission**				
** Yes**	Reference	–	Reference	–
** No**	.035 (.005 – .263)	.001	.279 (.028 – 2.798)	.278
** Unknown**	–	1.000	–	–
**Central vascular catheter**				
** Yes**	Reference	–	–	–
** No**	–	.999	–	–
**Peripheral vascular catheter**				
** Yes**	Reference	–	Reference	–
** No**	.120 (.056 – .258)	<.001	.163 (.063 – .426)	<.001
**Urinary catheter**				
** Yes**	Reference	–	Reference	–
** No**	.041 (.005 – .304)	.002	–	.997^*^
** Unknown**	–	.999	–	.999^*^
**Intubation**				
** Yes**	Reference	–	–	–^*^
** No**	–	.999	–	–^*^
**Hospitalization within 90 days**				
** Yes**	Reference	–	–	–^*^
** No**	.632 (.204 – 1.953)	.425	–	–^*^
** Unknown**	.381 (.028 – 5.274)	.472	–	–^*^

Note.^*^Estimates indicated by (–) could not be reliably calculated because of very small subgroup counts, resulting in unstable odds ratios and infinite confidence intervals (∞).

## Discussion

AMR is a critical global health challenge driven mainly by inappropriate antibiotic use in clinical settings. Understanding antibiotic prescribing patterns through hospital-based surveillance is essential for informing stewardship strategies.

In this study, among inpatients at Tanga RRH in Tanzania, the prevalence of antibiotic use was higher (77.6%) than that reported in previous studies from Tanzanian hospitals, with a range of 44% to 62.3% across the six referral hospitals.^11^ Similar high rates have been observed in Uganda (73.7%)^14^ and Kenya (62%).^15^ However, the rate at Tanga RRH is lower than that reported in some West African countries.^16^ Compared to middle-income countries such as Thailand^17^ and HICs such as Canada and Europe,^18^ the prevalence observed in Tanga RRH was markedly higher. These findings underscore the persistent global disparities in antibiotic use.^5–9^ Such regional differences likely reflect variations in AMS capacity, diagnostic infrastructure, regulation of prescribing, and the overall infectious disease burden. Stronger oversight and access to diagnostics in HICs help contain use, while limited resources and greater reliance on empirical therapy contribute to higher use in LMICs.

Older patients and those in surgical wards showed greater reliance on antibiotics, consistent with the clinical expectations and demands of these settings, where surgical procedures and empirical treatments are frequently employed. Similar ward-specific trends have been reported in Tanzanian hospitals, where pediatric and surgical patients are often prescribed antibiotics.^11^ Likewise, in Kenya, most pediatric inpatients received antibiotics,^19^ reinforcing the notion that empiric antibiotic use in high-risk groups is a common regional practice. Beyond these groups, cancer patients and other immunocompromised individuals are also recognized as high-risk populations, in whom frequent antibiotic use is often justified by increased susceptibility to infections.

Broad-spectrum antibiotics and pharmacological classes such as imidazoles, cephalosporins, and penicillins collectively dominate prescribing patterns. Prescribing was skewed toward Watch antibiotics, with Access agents falling short of the WHO target of 60% use.^20,21^ This overreliance on broad-spectrum agents such as ceftriaxone, a Watch antibiotic listed in both WHO AWaRe and Tanzania’s NEMLIT, raises concern, particularly in the absence of routine AST to guide use.

These findings align with those of previous studies in Tanzania and other African countries that have documented similar trends in prescription patterns.^22–24^ However, antibiotic use patterns vary globally. For example, cefotaxime and amoxicillin are predominant in Botswana, ampicillin and gentamicin in the Republic of Congo,^25^ piperacillin-tazobactam and cefazolin in Canada,^26^ and penicillin–beta-lactamase inhibitor combinations in Europe.^27^ These variations may reflect differences in disease burden and drug availability, as demonstrated by multi-country analyses of antimicrobial prescription patterns.^28,29^ In many African settings, the wide availability and low cost of drugs such as metronidazole and ceftriaxone contribute to their frequent use.^30^ Without AST data to support these prescriptions, the continued use of Watch Group antibiotics increases the risk of accelerating AMR.^20,29^

Despite the high prevalence of antibiotic use, microbiological testing to guide therapy is rarely performed. Prescribing seldom relies on laboratory results, reflecting broader patterns of dependence on empirical treatment in Tanzania due to diagnostic constraints.^11^ Similar trends of limited microbiological support for prescribing have been documented in Botswana, Ghana, and Sierra Leone.^31,32^ Several barriers may contribute to the underutilization of microbiology services, including clinicians not requesting tests, high costs, stockouts of reagents, incomplete patient records, and a lack of skilled personnel. As a result, empirical antibiotic prescribing remains the norm at Tanga RRH, increasing the risk of inappropriate use and driving AMR.^14^ Particularly in surgical wards, the routine practice of administering antibiotics before and after procedures—often in the absence of clear prophylactic guidelines—reflects a reliance on empirical treatment to mitigate infection risk, a phenomenon also reported in other African settings.^33,34^ Similar practices have been observed in the management of low-birthweight neonates and postpartum women.^35^ Strategic interventions are needed to address the underutilization of microbiology services. Strengthening the laboratory infrastructure, ensuring a regular supply of reagents, and building personnel capacity are critical steps. Integrating AST into clinical workflows through antibiotic stewardship programs can help clinicians move from empirical to evidence-based prescribing.

Our finding that only peripheral vascular catheter use remained independently associated suggests the importance of vascular access in driving prescription practices at Tanga RRH. This finding aligns with previous reports that patients with indwelling vascular devices are perceived to be at a higher risk of infection, leading clinicians to initiate antibiotics empirically as a preventive measure.^36–38^ Although admission to surgical and mixed wards, as well as surgery since admission, showed strong associations in the univariate analysis, these factors did not retain significance after adjustment. This suggests that their effect may be mediated through device-related exposures or the clinical context, rather than being independent predictors of outcomes. Similar patterns have been observed in other Tanzanian settings, where surgical patients frequently receive antibiotics; however, the influence diminishes once device use and ward-level factors are taken into account.^39^ Our findings suggest that surgery per se may not be an independent predictor of antibiotic use once device-related exposure and ward-level factors are considered. This highlights the importance of AMS interventions in prioritizing device management and ward-level prescribing practices, rather than focusing solely on surgical status.

Despite apparent alignment with national treatment guidelines, prescribing diverged from WHO stewardship priorities by leaning heavily toward Watch antibiotics rather than Access drugs. Similar gaps in adherence have been documented in Tanzanian primary healthcare settings and during earlier phases of stewardship implementation, underscoring the ongoing challenge of aligning practice with policy.^10,40^ These findings suggest an insufficient alignment with stewardship principles. Prioritizing narrow-spectrum *Access* antibiotics where clinically appropriate is a core stewardship strategy, as these agents carry lower resistance potential compared with broad-spectrum *Watch* and *Reserve* agents. Therefore, strengthening the diagnostic capacity to support de-escalation and targeted prescribing is essential to curb AMR in this setting.^20,21^

Taken together, the high prevalence of antibiotic use, low reliance on AST, and disproportionate use of Watch antibiotics underscore the urgent need to strengthen AMS at Tanga RRH. Interventions should include enforcing adherence to national guidelines, improving prescriber training, and addressing structural barriers, such as the lack of laboratory capacity and timely AST results. Integrating routine AST into clinical decision-making, particularly in surgical and high-risk wards, could curb empirical overuse. Furthermore, periodic PPSs and audits can help monitor antibiotic use patterns and guide policy adjustments aligned with WHO stewardship targets.

The limitations of this PPS include its single-site scope, which restricts generalizability to other hospitals in Tanzania, and the relatively small sample size. Because data were collected at a single point in time, the study could not capture seasonal variations in infection patterns or changes in prescription over time. The observed association between peripheral catheter use and antibiotic prescription, while consistent with the global literature, should be interpreted cautiously, as it reflects empiric practice in the absence of microbiological diagnostics rather than a novel finding. Despite these limitations, this study offers valuable baseline insights into antibiotic use patterns at a RRH in Tanzania, utilizing the standardized WHO PPS methodology. This underscores the need for future multi-center or national PPSs, as well as repeated surveys linked to stewardship interventions.

This study noted the underutilization of microbiology laboratory services, suggesting a heavy dependence on empirical prescribing. These findings underscore the need to strengthen AMS by improving diagnostic capacity, promoting targeted therapy, and enhancing adherence to treatment guidelines. Collaboration with regional and national health authorities is essential to align hospital practices with national policies and address the systemic drivers of inappropriate antibiotic use. Further qualitative research, including interviews with prescribers, and expanded PPS in other facilities, is recommended to understand prescribing patterns better and scale up stewardship interventions.

## Data Availability

Research data supporting this publication are available upon request.
